# Milk exosome-derived miRNAs from water buffalo are implicated in immune response and metabolism process

**DOI:** 10.1186/s12917-020-02339-x

**Published:** 2020-04-29

**Authors:** Zujing Chen, Yueqin Xie, Junyi Luo, Ting Chen, Qianyun Xi, Yongliang Zhang, Jiajie Sun

**Affiliations:** 1 Guangdong Engineering & Research Center for Woody Fodder Plants, South China Agricultural University, Guangzhou, 510642 China; 2 College of Forestry and Landscape Architecture, South China Agricultural University, Guangzhou, 510642 Guangdong China; 3 College of Animal Science, Guangdong Provincial Key Laboratory of Animal Nutrition Control, National Engineering Research Center for Breeding Swine Industry, South China Agricultural University, Guangzhou, 510642 China

**Keywords:** Buffalo milk, Exosomal miRNA, Cell-cell communication, Immune and metabolism

## Abstract

**Background:**

Buffalo milk is rich in various nutritional components and bioactive substances that provide more essential health benefits to human body. Recently, exosome identified in the breast milk has been reported as a neotype nutrient and can mediate intercellular communication with exosomal miRNAs. In the present study, we therefore hypothesized that exosome-derived miRNAs from buffalo milk would play the potential physiological importance of consumption of buffalo milk.

**Results:**

We isolated exosomes from buffalo and cow milk samples that were obtained at mid-lactation period, and the exosomal miRNA profiles were then generated using miRNA-seq. In addition, miRNAomes of pig, human and panda milk exosomes were downloaded from GEO database. Finally, a total of 27 milk exosomal miRNA profiles that included 4 buffalo, 4 cow, 8 pig, 4 human and 7 panda were analyzed using the miRDeep2 program. A total of 558 unique miRNA candidates existed across all species, and the top 10 highly expressed miRNA were evolutionarily conserved across multiple species. Functional analysis revealed that these milk enriched miRNAs targeted 400 putative sites to modulate disease resistance, immune responsiveness and basic metabolism events. In addition, a total of 32 miRNAs in buffalo milk were significantly up-regulated compared with non-buffalo milks, while 16 were significantly down-regulated. Of interest, functional analysis showed that up-regulated miRNAs were mainly related to host metabolism processes, while the predicted functions of down-regulated miRNAs were enriched in immune response.

**Conclusion:**

In this study, we explored the exosomal miRNAome differences between milks of different animals, expanding the theoretical basis for potential applications of the miRNA-containing vesicles.

## Background

Water buffaloes are predominant dairy animals, contributing the most important source of 13% to the milk production worldwide [1]. In recent years, buffalo milk has received increasing research interest and investment in various countries, owing mainly to its attractive nutrient content. Compared with cow milk, buffalo milk is characterized by a rich composition with fat [2], proteins [3], amino acids [4], vitamins and minerals [5, 6], as well as a healthier (i.e. lower) concentration of cholesterol and higher magnitude of unsaturated fatty acids [7]. In addition to nutritional ingredients, increasing evidences have reported that a type of membrane-bound carriers termed as exosomes were identified in the breast milk of human [8, 9], cow [10, 11] and pig [12], which have recently been considered as major players in cell-cell communication [13]. Exosomes are 40–100 nm diameter vesicles that are widely present in almost all biological fluids [14]. Exosomes have been proposed to signal by binding to the recipient cell surface receptors or by internalisation with the cell membrane [15], potentially donating substantial amounts of exosomal miRNAs to the recipient tissue/cells and subsequently playing pivotal roles in the post-transcriptional regulation of gene expression [14]. Previous report showed that human breast milk exosomes with immune modulatory features were important for the development of the infant’s immune system [8]. Further, the roles of bovine milk exosomes have been proved as transporters of miRNAs for eliciting regulatory functions in the recipient cells [10]. Overall, we therefore hypothesized that buffalo milk exosomes also provided novel information on miRNA composition differing from other types of non-buffalo milks, and highlighted the unrealized physiological importance of exosomes on drinking behaviour.

## Results

In 27 sequencing libraries, there were 558 unique miRNA candidates sequenced across all species, and only 395 miRNAs which were identified at least in four libraries were considered further (Additional file 1). Of these, the top 10 highly expressed miRNA in four buffalo libraries, which accounted for 75.45 ± 1.18% of all aligned reads, were evolutionarily conserved across multiple species. These miRNAs represented four different miRNA families; miR-let-7 (bta-let-7a-5p, bta-let-7b, bta-let-7c, bta-let-7e and bta-let-7f), miR-30 (bta-miR-30a-5p, bta-miR-30d and bta-miR-30e-5p), miR-148 (bta-miR-148a), and miR-26 (bta-miR-26a). In addition, four of these miRNAs (miR-148a, miR-30a-5p, bta-miR-30d, bta-let-7c) shared common ranking as top 10 expressed miRNAs when parsing the sequence data based on different species (buffalo, cow, pig, human and panda milks). To further identify the potential function of the top expressed miRNAs, target prediction was performed using miRanda software. A total of 400 putative target sites for the top 10 expressed miRNAs were identified (Additional file 2), and then all of these target candidates were submitted for homology and functional annotation using an online version of the DAVID program. These target genes belonged to 5 KEGG annotated categories that significantly related to a wide variety of disease resistance, immune responsiveness and basic metabolism events, including microRNAs in cancer, lysosome, glycerophospholipid metabolism, proteoglycans in cancer, galactose metabolism and neurotrophin signaling pathway (Additional file 3).

**Fig. 1:**
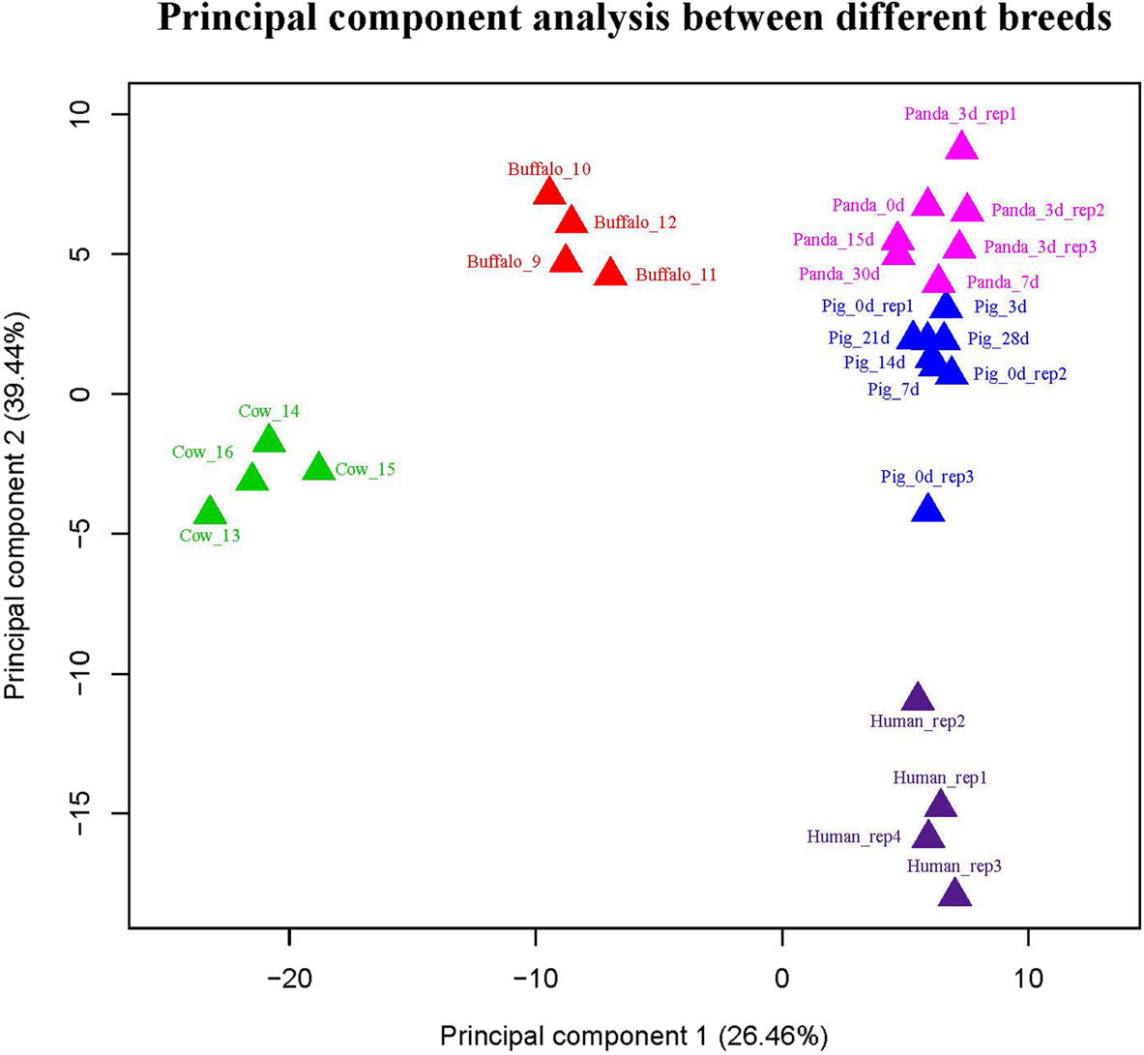
Principal component analysis of milk exosomal miRNAs between different species. The red, green, blue, purple and magenta represented for the expression of buffalo, cow, pig, human and panda milk exosomal miRNAome, respectively

**Fig. 2:**
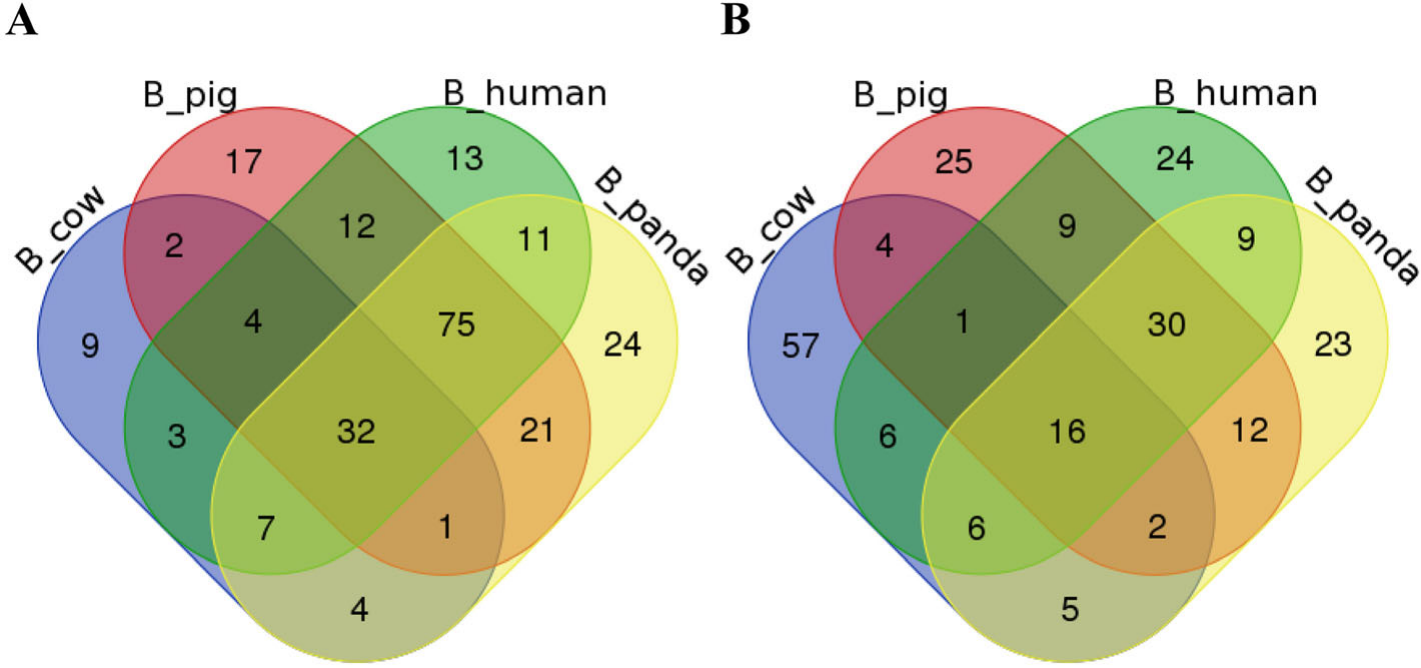
Differently up-regulated (**a**) and down-regulated (**b**) exosomal miRNAs between buffalo and non-buffalo milks. B_cow, B_pig, B_human and B_panda represented for the significantly expressed miRNAs between buffalo and cow, pig, human, panda, respectively

For validation and identification of species-specific miRNAs in buffalo milk, we compared the sequencing libraries between buffalo and non-buffalo milks and found that 27 indexed libraries were obviously divided into five groups based on breed differences (Fig. 1). To comparison with miRNA expression in non-buffalo milks, a total of 32 miRNAs in buffalo milk were significantly up-regulated (Fig. 2a), while 16 were significantly down-regulated (Fig. 2b) using the R Bioconductor package EdgeR analysis with TMM-normalized algorithm. In addition, target prediction revealed a total of 4372 miRNA-target interactions between significantly expressed miRNAs and NCBI RefSeq genes. This resulted in 1759 unique genes targeted by up-regulated miRNAs (Additional file 4), 1761 genes targeted by down-regulated miRNAs (Additional file 5), and 477 genes targeted by both up and down-regulated miRNAs. Especially, pathway analysis revealed that 39 predicted targets of up-regulated miRNAs were significantly enriched in categories annotated as a role in endocytosis (Additional file 6). On the contrary, the targets of down-regulated miRNAs were annotated with a total of 31 significantly KEGG pathways (Additional file 7).

## Discussion

In our sequencing libraries, miR-148a, miR-30a-5p, bta-miR-30d, bta-let-7c shared common ranking as top 10 expressed miRNAs when parsing the sequence data based on different species (buffalo, cow, pig, human and panda milks), suggesting these miRNAs could be important nutritional components of milk [16]. In details, the most abundant was bta-miR-148a accounting for 38.88 ± 3.12% in each buffalo library, which was consistent with previous reports in cow [17], pig [12] and human [9] milks. Recent characterizations of the human salivary exosome have also highlighted the miR-148a expression at high levels that expected to modulate oral cavity defence [18]. To date, although the underlying regulatory mechanism of the most predominant miRNA (miR-148a) packaged into milk exosomes was still unclear, it was intriguing to suggest that miR-148a was a potential biomarker for the quality control of mammalian milk [19]. The miR-30a was highly enriched in exosomes from the serum of acute myocardial infarction patients in vivo, and inhibition of miR-30a or exosome release contributed to maintaining of autophagy after hypoxia [20]. In addition to miR-30a, exosome-enriched miR-30d in endometrial fluid was proposed to be taken up by the pre-implantation embryo, thereby resulting in the observed modifications to the transcriptome and embryo adhesion [21]. The enrichment of let-7 miRNA family in the extracellular fractions, particularly, in the exosomes from gastric cancer cells reflected their oncogenic characteristics including tumorigenesis and metastasis [22]. These data implied the roles of highly expressed miRNAs with a specified function in the milk exosomes. Compared with non-buffalo milks, 32 miRNAs were significantly up-regulated, and 16 were down-regulated. Pathway analysis revealed that the targets of up-regulated miRNAs were significantly enriched in categories annotated as a role in endocytosis. The endocytosis pathway, for instance, has been highlighted as an important step in the delivery of bovine exosomes and their cargo to human vascular endothelial cells and peripheral tissues by endocytosis [23]. Additionally, we also revealed many of the other pathways which have been previously implicated in series basic metabolism processes in animals. These pathways included metabolic pathways enriched with 127 targets, glycerophospholipid metabolism enriched with 18 targets, protein processing in endoplasmic reticulum enriched with 26 targets, glycosaminoglycan biosynthesis enriched with 6 targets, biosynthesis of amino acids enriched with 13 targets, etc. Taken together, these analyses strongly suggested that up-regulated miRNAs that were differentially expressed between buffalo and non-buffalo milks were key regulators of the host metabolism processes shuttling within exosomes by endocytosis. In addition to metabolic pathways shared with the target annotation of up-regulated miRNAs, computational analysis of the down-regulated miRNA targets has identified the statistical over-representation of several terms previously implicated in response to immunity events, such as T cell receptor signaling pathway, NF-kappa B signaling pathway, AMPK signaling pathway, HTLV-I infection, lysosome, inflammatory mediator regulation of TRP channels, etc. The evidences indicated that the down-expressed miRNAs identified in this study were likely central regulators of the immune response and thus represented potential targets or novel biomarkers of infection and inflammation.

## Conclusions

Milk exosomal miRNA profiles that included 4 buffalo, 4 cow, 8 pig, 4 human and 7 panda were analyzed, and milk enriched miRNA candidates across all species were annotated to modulate disease resistance, immune responsiveness and basic metabolism events. This study provided an important mechanistic framework for future studies of dietary extracellular vesicles and the roles of milk miRNAs in human health and disease.

## Methods

### Milk exosomes collection and sequencing analysis

Buffalo (*Murrah* breed) and cow (*Friesian* breed) milk samples were obtained respectively from 4 healthy mid-lactating animals in a local farm (Panyu town, Guangzhou city, Guangdong province, China), and crude exosomes were purified following our previous method [24]. In brief, approximate 40 mL raw milk samples were subjected to 2 successive centrifugations at 2000 and 12,000×g at 4 °C for 30 min to remove somatic cells and debris. The supernatant was mixed with an equal volume of 0.25 M EDTA (pH 7.0) and incubated on ice for 15 min to precipitate casein and exosomes coated with casein. The suspension was ultracentrifuged at 80,000 g at 4 °C for 60 min (Sorvall WX Ultra 80, F37L-8 × 100 rotor; Thermo Scientific) to remove precipitated protein, milk fat globules, and microvesicles larger than exosomes. Exosomes were collected by centrifugation at 120,000 g at 4 °C for 90 min, resuspended in sterile phosphate-buffered saline, and filtered through a 0.22-μm membrane filter to obtain exosome solutions. Then, exosomal RNAs from each milk sample were used for library construction and subjected to single-end sequencing in the length of 40 nt with an Illumina Genome Analyzer (Biomarker Technologies, Beijing city, China). All procedures were conducted under protocols approved by the Institutional Animal Care and Use Committee of South China Agricultural University, China.

### MiRNA analysis

In addition to the indexed sequencing data of buffalos and cows, we downloaded the exosomal small RNA data of pig [12], human [8] and panda [25] in the GEO database (https://www.ncbi.nlm.nih.gov/gds/), respectively. Using the miRDeep2 program [17], a total of 27 sequencing libraries that included 4 buffalo, 4 cow, 8 pig, 4 human and 7 panda were further analyzed by blasting against bovine miRBase miRNA annotation (http://www.mirbase.org/) to identify conserved candidates between different species. The read count of each identified miRNA was firstly normalized with TMM-normalized algorithm, and the R Bioconductor package EdgeR analysis (http://bioconductor.org/packages/edgeR/) was applied to identify differentially expressed (DE) miRNAs with *P* value < 0.05 and fold change ≥2 between different groups.

### Target predictions and annotation

The 3′UTR sequences of the bovine RefSeq genes were downloaded from the University of California Santa Cruz (UCSC) table browser (http://genome.ucsc.edu/cgi-bin/hgTables). The targets of highly and differentially expressed miRNAs were successfully predicted using miRanda software (http://34.236.212.39/microrna/home.do), and the biological KEGG pathway analysis of the predicted targets were further performed by an online version of the DAVID program (https://david.ncifcrf.gov/).

## Supplementary information

**Additional file 1.** Exosomal miRNA expression between buffalo and non-buffalo milks. Buffalo_9, buffalo_10, buffalo_11 and buffalo_12 represented for 4 buffalo milk samples; cow_13, cow_14, cow_15 and cow_16 represented for 4 cow milk samples; pig_0_rep1, pig_0_rep2, pig_0_rep3, pig_3d, pig_7d, pig_14d, pig_21d and pig_28d represented for 8 pig milk samples; human_rep1, human_rep2, human_rep3 and human_rep4 represented for 4 human milk samples; panda_0d, panda_3d_rep1, panda_3d_rep2, panda_3d_rep3, panda_7d, panda_15d and panda_30d represented for 7 panda milk samples; logFC, log fold change; logCPM, log counts per million; FDR, false discovery rate.

**Additional file 2.** Predicted targets of top 10 expressed miRNAs in buffalo milk exosome. Query represented for miRNA candidates; Ref represented for reference genes.

**Additional file 3.** The KEGG analysis of top 10 expressed miRNAs’ targets

**Additional file 4.** Predicted targets of significantly up-regulated miRNAs in buffalo milk exosome

**Additional file 5.** Predicted targets of significantly down-regulated miRNAs in buffalo milk exosome

**Additional file 6.** The KEGG analysis of the up-regulated miRNAs’ targets

**Additional file 7.** The KEGG analysis of the down-regulated miRNAs’ targets

## Data Availability

The miRNA-seq raw datasets of buffalo and cow milk exosome have been deposited into NCBI SRA database with the BioProject accession number PRJNA606117. The exosomal small RNA datas of pig, human and panda were free downloaded from the publicly accessible NCBI GEO database with the Accession Nnumber GSE36590, GSE32253 and GSE89755, and this is an open escience data sharing platform and allowed to shared the datas for scientific research.

## Abbreviations

GEO: NCBI Gene Expression Omnibus
DAVID: The Database for Annotation, Visualization and Integrated Discovery
KEGG: Kyoto Encyclopedia of Genes and Genomes
TMM: the trimmed mean of M-values
AMPK: Adenosine 5′-monophosphate-activated protein kinase
